# Benign thyroid nodule unresponsive to radiofrequency ablation treated with laser ablation: a case report

**DOI:** 10.1186/s13256-018-1628-9

**Published:** 2018-05-11

**Authors:** Silvia Oddo, Margherita Balestra, Lara Vera, Massimo Giusti

**Affiliations:** Endocrinology Unit, Istituto di Ricovero e Cura a Carattere Scientifico (IRCCS), Azienda Ospedaliera Universitaria (AOU) San Martino University, Istituto Nazionale per la Ricerca sul Cancro (IST), Largo Rosanna Benzi, n°10, I-16132 Genoa, Italy

**Keywords:** Radiofrequency, Laser, Thyroid, Thyroid ablation, Thyroid nodules

## Abstract

**Background:**

Radiofrequency ablation and laser ablation are safe and effective techniques for reducing thyroid nodule volume, neck symptoms, and cosmetic complaints. Therapeutic success is defined as a nodule reduction > 50% between 6 and 12 months after the procedure, but a percentage of nodules inexplicably do not respond to thermal ablation.

**Case presentation:**

We describe the case of a young Caucasian woman with a solid benign thyroid nodule who refused surgery and who had undergone radiofrequency ablation in 2013. The nodule did not respond in terms of either volume reduction or improvement in neck symptoms. After 2 years, given the patient’s continued refusal of thyroidectomy, we proposed laser ablation. The nodule displayed a significant volume reduction (− 50% from radiofrequency ablation baseline volume, − 57% from laser ablation baseline), and the patient reported a significant improvement in neck symptoms (from 6/10 to 1/10 on a visual analogue scale).

**Conclusions:**

We conjecture that some benign thyroid nodules may be intrinsically resistant to necrosis when one specific ablation technique is used, but may respond to another technique. To the best of our knowledge, this is the first description of the effect of performing a different percutaneous ablation technique in a nodule that does not respond to radiofrequency ablation.

## Background

Since 2010, practical guidelines have approved radiofrequency ablation (RFA) and laser ablation (LA) as possible therapeutic options for the treatment of benign symptomatic thyroid nodules in patients who refuse thyroidectomy or in those with comorbidities that contraindicate surgery [1, 2]. RFA and LA are safe and effective techniques for reducing nodule volume, neck symptoms, and cosmetic complaints [3, 4].

Therapeutic success is defined as a nodule reduction > 50% from the baseline volume 6–12 months after the procedure and is reportedly achieved in 67% and 81% of cases of LA and RFA, respectively, in 6- to 12-month follow-up [3, 4]. However, for unknown reasons, a percentage of nodules do not respond to thermal ablation.

To the best of our knowledge, this report describes the first case in the literature of a benign thyroid nodule treated unsuccessfully with RFA in which subsequent LA treatment achieved a significant reduction in volume and neck symptoms. This report suggests that practitioners of minimally invasive thermal ablation can try different ablation techniques on thyroid nodules that do not respond to RFA.

## Case presentation

A 41-year-old Caucasian woman discovered the presence of a mass on neck palpation. Her family history was negative for thyroid illnesses, and she stated that she had no past medical history. The woman was an office worker, did not live or work in an iodine-poor area, and reported having a good dietary intake of iodine. She neither smoked nor drank alcohol and was not taking any medication. She was lean (body mass index 17 kg/m^2^), and her blood pressure, heart rate, and temperature were normal (100/65 mmHg, 76 beats/minute, and 36.2 °C, respectively). A physical examination confirmed the presence of a soft mass in the right portion of her neck, which was mobile on swallowing. The results of her cardiac, pulmonary, abdominal, and neurological examinations were unremarkable.

**Fig. 1:**
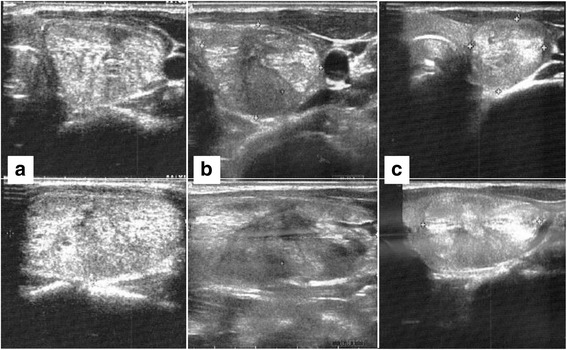
Ultrasound images showing the thyroid nodule (**a**) at the baseline, (**b**) 24 months after radiofrequency ablation, and (**c**) 12 months after laser ablation

The patient underwent an ultrasound (US) scan, which showed a single, solid, isoechoic nodule of 12 ml (anteroposterior × laterolateral × craniocaudal diameters 20 × 30 × 40 mm, respectively) in the right lobe of the thyroid (Fig. 1a). She was symptomatic for neck discomfort, with a score of 6/10 on a visual analogue scale. Her thyroid function was normal (thyroid-stimulating hormone concentration 1.81 mIU/L, free T3 5.0 pmol/L, free thyroxine 14.12 pmol/L); her calcitonin level was normal (< 1 ng/L); and her thyroid autoimmunity was negative (thyroid peroxidase antibodies 18 U/ml).

The patient underwent a fine-needle aspiration biopsy (FNAB), which revealed a colloid nodular hyperplasia with hyperplastic thyrocytes and fluid colloid (Thy2 according to the British Thyroid Association). Because she refused lobectomy for fear of surgery, we proposed RFA of the nodule. After a second FNAB (Thy2 according to the British Thyroid Association), a phoniatric evaluation, which excluded anomalies of vocal cord motility, and an electrocardiogram, which excluded arrhythmias as suggested by the Korean consensus statement [5], a single session of RFA was performed by means of transisthmic access and moving-shot technique [5], with a delivered energy of 41,868 J (3489 J/ml) in a day-hospital regimen. RFA was performed by means of 7-cm, 18-gauge electrodes with a 1-cm active tip, with the support of the MyLabFive US system (Esaote, Genoa, Italy) with a 7.5-MHz linear probe (LA523). The radiofrequency generator used was a Viva VRS01 RF system (STARmed, Seoul, Korea), and the peristaltic pump used was an R4S100 (STARmed, Seoul, Korea).

Before RFA, the patient underwent an intravenous infusion for 30 minutes of ketorolac (20 mg) and ranitidine (50 mg) diluted in 100 ml of 0.9% saline solution. Subsequently, intravenous ketorolac (40 mg) and ranitidine (50 mg) were administered in 500 ml of 0.9% saline for about 5 hours (during and after thyroid RFA). Preprocedural local anesthesia with 2% lidocaine was carried out at the puncture site. The procedure was well tolerated by the patient, and no adverse events occurred. After thyroid RFA, a compressive bandage and ice were applied to the patient’s neck, and she received a domiciliary prescription for steroid administration (prednisone 25 mg for 3 days, 12.5 mg for 3 days, 6.25 mg for 3 days) and gastric protection. At the time of the RFA, our operator had 1 year of experience with this technique.

**Fig. 2:**
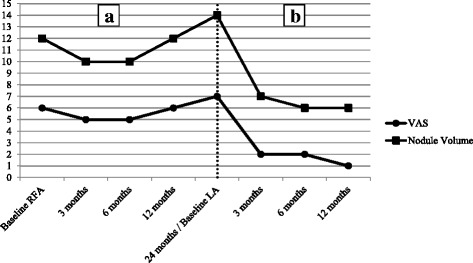
Changes in the volume of the nodule and in neck symptoms. (**a**) Changes in volume and in neck symptoms, as indicated on the visual analogue scale, from baseline to 24 months after radiofrequency ablation. (**b**) Changes in volume and in neck symptoms, as indicated on the visual analogue scale, from baseline of laser ablation (24 months after radiofrequency ablation) to 12 months after laser ablation. RFA Radiofrequency ablation, VAS Visual analogue scale

At both the 6- and 12-month follow-up examinations, the patient’s neck discomfort was seen to persist (Fig. 2a). A physical examination confirmed the unchanged presence of the soft mass, which was mobile on swallowing, in the right portion of the neck. On a US scan, the nodule displayed only a modest and transitory volume reduction up to the 6th month, followed by regrowth (Figs. 1b and 2a).

Twenty-four months after RFA, because the patient again refused to undergo lobectomy, we proposed a single session of LA. This was performed by the same operator, who had 1 year of experience with this technique, by means of two 300-μm-diameter fibers inserted by means of a 21-gauge Chiba needle (Elesta, Calenzano, Italy) with one pull-back of 1 cm and a delivered energy of 5489 J (457 J/ml). LA was performed by means of a commercially available US scanner (EchoLaser X4®; Esaote, Genoa, Italy) equipped with a 7.5-MHz linear transducer (LA 332; Esaote, Genoa, Italy) with a 1064-μm diode laser unit and an individual energy emission setting and independent activation. The preprocedural modalities were the same as for RFA.

The patient tolerated the procedure well, and no adverse events occurred. At the time of LA, our operator had 1 year of experience with this technique. The patient reported a progressive improvement in neck symptoms and cosmetic complaints, and follow-up examinations showed a marked, progressive reduction in nodule volume (Figs. 1c and 2b). The patient’s thyroid function and thyroid autoimmunity remained unchanged after RFA and LA (Table 1).

**Table 1:** thyroid function and autoimmunity at each time-point of the study

	TSH	f-T3	f-T4	Calcitonin	TPO-Abs
	(mIU/L)	(pmol/L)	(pmol/L)	(ng/L)	(U/mL)
	(normal values 0.27-4.2)	(normal values 2.76-7.07)	(normal values 11.97-21.88)	(normal values <10)	(normal values <100)
Baseline RFA	1.81	5.00	14.12	<1	18
3rd month	1.60	4.98	15.30	-	19
6th month	1.43	5.20	14.35	-	25
12th month	1.57	5.05	15.07	-	15
24th month/Baseline LA	1.61	5.30	14.78	-	26
3rd month	1.47	5.12	15.03	-	30
6th month	1.36	4.97	14.99	-	24
12th month	1.89	5.00	15.12	-	19

## Discussion

We report a case of a young woman who underwent RFA for treatment of a symptomatic benign nodule of the right lobe of the thyroid after refusing thyroidectomy. After 24 months, she underwent LA on the same nodule because RFA had failed to reduce the volume of the nodule and to alleviate her neck discomfort. To the best of our knowledge, this is the first description of the effect of performing a different percutaneous ablation technique in a nodule that did not respond to RFA.

Both RFA and LA are percutaneous techniques, and their efficacy in reducing thyroid nodule volume is reported to be similar [6]. Therapeutic success (nodule volume reduction > 50%) is achieved in the majority of cases by both techniques [3, 4].

Some studies have identified factors that might predict a good response to RFA, though these are controversial: small volume (< 12 ml) of the nodule at baseline [7], the absence of vascularization [8], the presence of a fluid component [9], nonfunctioning status [10], and the presence of well-defined margins [4]. By contrast, Papini *et al*. [3] found that baseline size, presence of goiter or US findings (such as fluid component, halo, vascularization, and calcifications) were not predictive of a volume decrease > 50% in thyroid nodules treated with LA. Our patient’s thyroid nodule was < 13 ml, nonfunctioning, well-defined, and free from vascularization; thus, there were no parameters that could predict the inefficacy of RFA.

When therapy is not successful, another session of ablation may be proposed; in the second session, the same ablation procedure is generally performed [11]. This choice is probably determined by the availability of only LA or RFA in each center. In our center, however, we had the possibility to perform both RFA and LA. We chose to perform LA after unsuccessful RFA because, in our cohort of patients, we had already observed two cases in which the repetition of RFA had failed to yield significant results in nodules that had previously been treated with an unsuccessful RFA procedure. We therefore conjectured that certain nodules might be intrinsically resistant to necrosis when one ablation technique is undertaken, but might respond to the other ablation technique. Indeed, in our patient, the thyroid nodule, which had not responded to a single session of RFA, displayed an optimal response to a single session of LA. Differences in response to one or the other technique might also depend on the operator’s experience. In our patient, however, the same operator performed RFA and LA and had the same amount of experience (1 year) in performing both procedures. We therefore excluded the possibility that the better response of the nodule to LA might have been due to greater operator experience with this technique.

## Conclusions

In our opinion, LA may be a viable option in patients with a thyroid nodule that does not respond to RFA and who refuse surgery or have comorbidities that contraindicate it. It would be useful to conduct a study with a large cohort of patients with an inefficient response to one of these techniques who are subsequently treated with the other technique.

## Abbreviations

FNAB: Fine-needle aspiration biopsy
LA: Laser ablation
RFA: Radiofrequency ablation
f-T3: Free triiodothyronine
f-T4: Free thyroxine
TPO: Thyroid peroxidase
TSH: Thyroid-stimulating hormone
US: Ultrasound
